# Knowledge and perceptions of physicians from private medical centres towards generic medicines: a nationwide survey from Malaysia

**DOI:** 10.1186/s40545-015-0031-9

**Published:** 2015-03-16

**Authors:** Rohit Kumar, Mohamed Azmi Hassali, Fahad Saleem, Alian A Alrasheedy, Navneet Kaur, Zhi Yen Wong, Muhamad Ali SK Abdul Kader

**Affiliations:** Discipline of Administrative and Social Pharmacy, School of Pharmaceutical Sciences, Universiti Sains Malaysia, Penang, 11800 Malaysia; Pharmacy Practice Department, College of Pharmacy, Qassim University, Qassim, Saudi Arabia; Discipline of Physiology and Pharmacology, School of Pharmaceutical Sciences, Universiti Sains Malaysia, Penang, 11800 Malaysia; Pharmacy Department, Hospital Teluk, Intan, Perak Malaysia; Department of Cardiology, Hospital Pulau Pinang, Jalan Residensi, 10990 Penang Malaysia

**Keywords:** Generics, Quantitative, Perceptions, Malaysia, Private hospitals

## Abstract

**Objectives:**

Generic medicine prescribing has become a common practice in public hospitals. However, the trend in private medical centres seems to be different. The objective of this study was to investigate knowledge, perceptions and behavior of physicians from private medical centres in Malaysia regarding generic medicines.

**Methods:**

This study was a cross-sectional nationwide survey targeting physicians from private medical centres in Malaysia. The survey was conducted using questionnaire having (i) background and demographic data of the physicians, volume of prescription in a day, stock of generic medicines in their hospital pharmacy etc. (ii) their knowledge about bioequivalence (iii) prescribing behavior (iv) physicians’ knowledge of quality, safety and efficacy of generic medicines, and their cost (v) perceptions of physicians towards issues pertaining to generic medicines utilization.

**Results:**

A total of 263 questionnaires out of 735 were received, giving a response rate of 35.8%. Of the respondents, 214 (81.4%) were male and 49 (18.6%) were females. The majority of the participants were in the age range of 41–50 years and comprised 49.0% of the respondents. Only 2.3% of physicians were aware of the regulatory limits of bioequivalence standards in Malaysia. Of the respondents, 23.2% agreed that they ‘always’ write their prescriptions using originator product name whereas 50.2% do it ‘usually’. A number of significant associations were found between their knowledge, perceptions about generic medicines and their demographic characteristics.

**Conclusions:**

The majority of the physicians from private medical centres in Malaysia had negative perceptions about safety, quality and the efficacy of generic medicines. These negative perceptions could be the cause of the limited use of generic medicines in the private medical centres. Therefore, in order to facilitate their use, it is recommended that the physicians need to be reassured and educated about the drug regulatory authority approval system of generic medicines with regard to their bioequivalence, quality, efficacy and safety. Apart from the policy on generic substitution, it would also be recommended to have a national medicine pricing policy, which controls drug prices, in both the public and private sector. These efforts are worthwhile to reduce the drug expenditure and improve the medicine affordability in Malaysia.

## Introduction

Malaysia has built a complex, but comprehensive healthcare system, that keeps a tab on every level of healthcare related service in the country, from medical institutions to the primary end user–the patient [1]. Malaysia has a comprehensive two-tiered healthcare system that consists of a government-run public sector and a private healthcare sector. The public sector is heavily subsidised by the government and represents about 70% of the healthcare services and while the private sector is limited to those who can afford it and represents about 30% of the healthcare services [2]. In the public sector, the Ministry of health (MOH) is the main government body accountable for providing healthcare services in the country [3]. The private sector offers both remedial and rehabilitation services and is financed strictly on a fee-for-service basis. The cost is paid fully by patients themselves, their employers, and/or by their insurance companies [3,4]. Despite their short history, private-for-profit hospitals have been increasing at a very fast pace in most developing countries including Malaysia [5]. To date, the private healthcare providers dominate the market, and a total of 62% of all hospitals are owned by private entities [6]. This increasing demand is due to an increase in income per capita following the deregulation in the supply of healthcare services, an increase in the awareness on the importance of healthcare, and access to the Internet, resulting in an increased understanding on the importance of the early treatment for critical diseases [6]. In comparison to the public sector, these hospitals have acquired state-of-the art machines, best talent pool, English speaking staff, medical tourism promotional packages and above all government has provided them incentives for expenses incurred in obtaining international accreditations etc. [6,7]. All of these efforts have today resulted in more than 225 private hospitals which are expected to grow to 239 by 2018 [7].

With this insight into the Malaysian healthcare system, it is prudent to know the financial side of this sector with a special attention to private healthcare system. In tandem, Malaysia’s healthcare expenditure is estimated to reach nearly US$ 20.4 billion (bn) by 2018 [8]. The Total Health Expenditure (TEH, nominal) for Malaysia during 1997–2012 ranged from US$ 2.67bn in 1997 to US$ 13.65bn in 2012. During the period of 1997–2012, the share of private expenditure in overall healthcare expenditure rose to 47% [9]. In 2013, private healthcare expenditure reached US$ 6.47bn, representing 53.5% of the total healthcare outlay in Malaysia [8]. Like most of the developing countries, Malaysia does not have a national social health insurance scheme. Private health insurance is voluntary and is generally taken out mainly to cover private health services. Private household out-of-pocket payments (OOPPs) form the largest component of private healthcare spendings [10,11]. In 2009, Malaysia’s out-of-pocket (OOP) spending was 77% of private sector spending, which is twice the high-income country OOP average at 37% of private sector spending [12,13]. The OOPPs contribution has gone up to an average of 79% of the private sector’s expenditure in 2012 [9,14]. The contribution of the expense on pharmaceuticals out of this 79% OOPPs is not known. These OOPPs are set to worsen as price increases are proposed both in terms of specialist doctors’ fees and the prospect of pharmacists charging for consultations [8]. One of the best documented barriers to medication adherence is high OOP costs even among individuals with prescription drug insurance [15]. The government hospitals currently dispenses most drugs free of-charge, but patients who use private sector pay for their medicine and these costs can be substantial. Costs of medicines in the private sector of Malaysia are higher than in the public sector. As dispensing doctors and private retail pharmacies apply high mark-up prices, the practice of price control and the greater use of generic medicines are required to make the medicines affordable to Malaysian public [16]. Above all, medicine prices in the private market of Malaysia are determined by market force, without any control by the government. There is no national drug pricing policy exists and studies have reported that the prices of medicines are high in Malaysia [17-19].

To bring down the OOPPs and reduce financial burden on patients in private healthcare sector, it is highly recommended to promote use of generic medicines [20]. This can allow considerable savings in health care cost without affecting the quality or the therapeutic effect of the prescribed medicine and will also reduce OOPPs burden substantially [21-24]. The acceptance of generic medicines in medical practices is a complex phenomenon and many factors can influence it. Physicians play an important role in controlling this phenomenon and their decision in prescribing generic drugs is likely to be affected by many determinants [25]. Although there is increasing local and international encouragement for physicians to prescribe generic medicines, some physicians are not in favor of prescribing them. Therefore, many studies have tried to find determinants of this practice [26-28]. In Malaysia, generic drug prescribing has become a common practice in public hospitals. However, there is a paucity of data regarding the perspectives of Malaysian private medical practitioners regarding generic medicines. Therefore, the aim of this study was to investigate knowledge, perception and behavior of physicians from private medical centres of Malaysia regarding generic medicines and to examine factors that affect their prescribing pattern regarding choice of medicine brands (i.e. generic medicines and innovator medicines).

## Methods

### Study design: the study instrument/questionnaire

This survey was conducted using a self-completed anonymous questionnaire which was adopted and modified from a previous study conducted by Chua, et al. [29]. The questionnaire was validated using an expert review and a field evaluation by potential respondents [30,31]. The questionnaire was designed in order to elicit knowledge, perception, and behaviour of physicians towards generic medicines. It contained 10 multiple-choice questions and an 18 Five-Point Likert Scale statements and comprised 5 sections: (i) the background and the demographic data of the physicians, including their experience, their volume of prescriptions in a day, and the stock of generic medicines in their hospital pharmacy, etc. (ii) their knowledge about bioequivalence (iii) prescription behaviour (iv) the physician’s knowledge and beliefs regarding quality, safety and efficacy of generic medicines, together with their cost in comparison to innovator products (v) the perceptions of the prescribers to issues pertaining to generic medicine utilisation in Malaysia.

### Study participants and sampling

This nationwide survey targeted physicians working at the private medical centres in Malaysia. Since the study population was private medical centres, the member hospitals registered with the Association of Private Hospitals of Malaysia (APHM) as of May 2013, were considered as potential participants. Out of 114 hospitals registered with the APHM, 95 hospitals were contacted during the period of May 2013–June 2013. A total of 19 Hospitals having less than 15 beds, and centres which only provide maternity services were excluded from this study.

### Survey administration

First of all, before conducting the survey, a letter of introduction was sent to the President of the APHM, seeking approval for the study, and informing its members of the planned survey, and to prepare the potential respondents for the survey. Then, along with the approval letter from the APHM, the final validated survey questionnaire, with a cover letter were sent through ordinary mail, or electronic mail, to the Human Resources Department (HRD), or the person in charge, of all 95 hospitals. The cover letter included a request for their willingness to participate in the survey, explaining the importance and the purpose of the study. Follow-up telephone calls and electronic mail reminders were sent to non-respondents. There was no response from 39 hospitals and 22 institutions declined to participate in the study. The institutions that agreed to participate permitted us to contact the physicians directly, or through their HRD. The required copies of the questionnaire were posted to the contact person of the participating hospitals. The completed survey forms were collected by the HRD of the hospitals, and sent to the researchers through ordinary or electronic mail. However, for some hospitals, the survey forms were distributed and collected by the researchers.

### Ethical consideration

Ethical approval for this study was obtained from the ethical committee named “the Joint Ethics Committee of the School of Pharmaceutical Sciences, USM–Hospital Lam Wah Ee on Clinical Studies”, Penang, Malaysia (USM-HLWE/IEC/2013(0004). Participation in the study was voluntary and no compensation was paid to the participants. The information received was anonymous and treated confidentially.

### Statistical analysis

The survey questionnaire was categorised into five responses (Strongly Agree=5, Agree=4, Neutral=3, Disagree=2, and Strongly Disagree=1). The frequency and the percentages were used for the descriptive analyses to examine the characteristics of the respondents and the responses for each question in the survey [32]. In order to examine the associations between the physician’s background, and the variables related to their knowledge about generic medicines were assessed by using Mann Whitney and Kruskal Wallis tests. All statistical tests were conducted at an *a priori* significance level of *p*<0.05 using Statistical Package for the Social Sciences (SPSS) Version 16 for Windows.

## Results

### Demographic and practice characteristics

A total of 263 questionnaires out of 735 were received by researchers from those hospitals who agreed to participate in the study, giving a response rate of 35.8%. However, this is the overall response rate, because the effective response rate could not be measured as the survey form distribution and the collection was handled directly by the HRDs of the many hospitals. Among all of the respondents, 214 (81.4%) were males and 49 (18.6%) were females. The majority of the participants were in the age range of 41–50 years and comprised 49.0% of the whole respondents. This age group of participants graduated between the years 1991–2000. 51.7% of the prescribers had a postgraduate degree and 55.5% were graduated from Malaysia. 30.4% of the physicians had been practising for more than 25 years and 39.9% of the subjects had a prescription rate of 11–20 prescriptions per day. Almost 57.0% of the respondents informed that the stock of generic medicine in their hospital pharmacy was less than 10%, whereas 29.3% of the respondents mentioned that this availability was between 11%–40%. The stock of generic medicines in the hospital pharmacies was more than 61%, according to 10.6% of the cases only. The detailed demographic characteristics are presented in Table 1.

**Table 1:** **Demographic and practice characteristics of participants**

**Variables**	**n**	**%**
Sex		
Male	214	81.4
Female	49	18.6
Age Range (years)		
Under 30	4	1.5
30–40	46	17.5
41–50	129	49.0
51–60	54	20.5
61–70	30	11.4
Year of graduation		
Before 1980	40	15.2
1981–1990	69	26.2
1991–2000	130	49.4
2001–2005	13	4.9
2006–2010	11	4.2
Place of graduation/specialization*		
Malaysia	146	55.5
India	53	20.2
UK	29	11.0
Australia	19	7.2
Others	16	6.1
Postgraduate degree		
Yes	127	48.3
No	136	51.7
Experience (years)		
≤5	5	1.9
6–10	18	6.8
11–15	57	21.7
16–20	66	25.1
21–25	37	14.1
>25	80	30.4
Number of Prescriptions/day		
≤10	67	25.5
11–20	105	39.9
21–39	68	25.9
≥40	23	8.7
% of generics stock in hospital pharmacy		
≤10	150	57.0
11–40	77	29.3
41–60	28	10.6
≥61	8	3.0

*Place of highest level of education was considered.

### The doctor’s knowledge about the regulatory bioequivalence standards for generic drug products

The physicians were requested to tick on the relevant answer to a multiple choice question to identify the Malaysia’s National Pharmaceutical Control Board (NPCB) bioequivalence standard. An introductory statement was used to explain the question as follows: ‘The NPCB, Malaysia considers a product to be bioequivalent if its bioavailability is within a specified range compared with the currently marketed branded product. The regulatory limits applied are that 90% confidence intervals for the ratios (generic product: brand name product) of the areas under the plasma drug concentration versus time curves and the maximum plasma drug concentrations must fall between?’ The results showed that only 2.3% of the respondents could answer it correctly.

### Prescribing behaviour of physicians

In the questions related to prescribing behaviour, 23.6% of the respondents agreed that they ‘always’ write their prescriptions using originator product names, whereas 49.4% of the physicians do it ‘usually’. Only 9.9% of the physicians indicated that their prescription was ‘sometimes’ a brand name.

### The knowledge about generic medicines

The responses received and the influences of demographic characteristics on physicians*’* knowledge about generic medicines are provided in Tables 2 and 3 respectively. The possible associations between the beliefs about generic medicines and the demographic characteristics were assessed using Mann Whitney and Kruskal Wallis Tests, wherever applicable.

**Table 2:** **Knowledge about generic medicines**

**Item description**	**n (%)**				
	**Strongly disagree**	**Disagree**	**Neutral**	**Agree**	**Strongly agree**
A generic medicine is bioequivalent to a brand name medicine.	2 (0.8)	46 (17.5)	80 (30.4)	130 (49.4)	5 (1.9)
A generic medicine must be in the same dosage form (e.g. tablet, capsule) as the brand name medicine.	0 (0.0)	30 (11.4)	47 (17.9)	146 (55.5)	40 (15.2)
A generic medicine must contain the same dose as the brand name medicine.	0 (0.0)	16 (6.1)	26 (9.9)	159 (60.5)	62 (23.6)
Generic medicines are less effective compared to brand name medicines. (r)	6 (2.3)	49 (18.6)	72 (27.4)	93 (35.4)	43 (16.3)
Generic medicines produce more side effects compared to brand name medicines. (r)	8 (3.0)	103 (39.2)	100 (38.0)	45 (17.1)	7 (2.7)
Brand name medicines are required to meet higher standards than generic medicines. (r)	5 (1.9)	31 (11.8)	32 (12.2)	137 (52.1)	58 (22.1)
There are too many generic brands available.	0 (0.0)	15 (5.7)	35 (13.3)	154 (58.6)	59 (22.4)
Generics are cheaper for patients than original brands.	0 (0.0)	0 (0.0)	7 (2.7)	123 (46.8)	133 (50.6)
It is easier to remember brand names, rather than generic drug names.	3 (1.1)	39 (14.8)	39 (14.8)	139 (52.9)	43 (16.3)
Patients prefer original brands, they get confused with generics. (r)	1 (0.4)	42 (16.0)	54 (20.5)	143 (54.4)	23 (8.7)
Pharmacists should dispense generic brands, if patient agrees.	21 (8.0)	76 (28.9)	68 (25.9)	91 (34.6)	7 (2.7)

**Table 3:** **Comparison between demographic characteristics and knowledge towards generic medicines**

**Item description**	Gender*	Age**	Year of graduation**	Postgraduate qualification*	Country of graduation**	Year of practice**	No. of prescriptions written/day**	% of generics in stock**
A generic medicine is bioequivalent to a brand name medicine.	0.240	0.764	0.906	0.244	0.322	0.366	0.005	0.285
A generic medicine must be in the same dosage form (e.g. tablet, capsule) as the brand name medicine.	0.085	0.491	0.390	0.572	0.717	0.595	0.251	0.174
A generic medicine must contain the same dose as the brand name medicine.	**0.039**	0.503	0.220	0.327	0.450	0.461	**0.048** ^**a**^	0.261
Generic medicines are less effective compared to brand name medicines. (r)	0.265	0.239	0.915	0.100	0.436	**0.047**	**0.003** ^**a**^	**0.000** ^**a**^
Generic medicines produce more side effects compared to brand name medicines. (r)	0.535	**0.001** ^**a**^	0.058	0.974	0.777	0.087	**0.041**	0.046
Brand name medicines are required to meet higher standards than generic medicines. (r)	0.510	**0.050**	0.559	0.120	0.426	**0.046**	0.144	0.147
There are too many generic brands available.	0.105	**< 0.001** ^**a**^	0.084	0.207	0.541	**< 0.001** ^**a**^	0.019	0.452
Generics are cheaper for patients than original brands.	0.183	0.418	0.396	0.077	0.360	0.213	0.274	**0.029**
It is easier to remember brand names, rather than generic drug names.	**0.041**	**0.005** ^**a**^	**0.007**	0.629	0.150	**< 0.001** ^**a**^	**0.018** ^**a**^	**< 0.001** ^**a**^
Patients prefer original brands, they get confused with generics. (r)	0.171	0.385	0.883	0.930	0.293	0.573	0.117	**0.002** ^**a**^
Pharmacists should dispense generic brands, if patient agrees.	0.221	0.121	0.099	0.921	0.413	0.598	0.420	**0.028**

(r): Reversed ítem. *Mann Whitney test, **Kruskal Wallis test was used. P < 0.05 is considered significant. ^**a**^Significant after Bonferroni correction.

Bold values indicate statistical significance.

A Likert-type scale was used to evaluate the respondent’s knowledge about generic medicines being bioequivalent to their reference product. Although only 2.3% of the physicians were aware of the regulatory limits of the bioequivalence standard in Malaysia, interestingly, 51.3% of the respondents believed that generic products were bioequivalent to their branded counterparts. Forty six (17.5%) of the respondents disagreed with this statement, and the rest remained neutral. The majority of the participants, 70.7% and 84.1%, mentioned that generic medicines should be in same dosage form, and should have same dose strength as the branded product, respectively. A significant association was noted between the number of prescriptions written per day, and the physicians response to the statement that generic medicines should contain the same dose as the branded product, [H(3)=7.898, p=0.048). The Bonferroni correction at 0.0083 level of significance revealed that the difference was between the respondents who wrote less than 10 prescriptions per day as they expressed stronger agreement to this statement when compared to the physicians who wrote 21–39 prescriptions per day (U=1743.50, r=−0.17, p=0.007).

51.7% of the physicians stated that generic medicines are less effective when compared to their reference product, whereas 27.4% of the respondents remained neutral and 20.9% disagreed with this statement. A significant association was found among the number of prescriptions written per day, the percentage of generic medicines in stock, and the physician’s views on the low effectiveness of generic medicines when compared to branded products [H(3)=13.973, p=0.003] and [H(3)=25.179, p<0.0001], respectively. For the number of prescriptions written per day, a Bonferroni correction at a 0.0083 level of significance revealed that the respondents who wrote < 10 prescriptions per day, expressed a stronger agreement to the statement, when compared to the doctors who wrote > 40 prescriptions per day [U=1541.00, r=−0.21, p=0.001] and 21–39 prescriptions per day [U=488.50, r=−0.17, p=0.006], respectively. The physicians working within hospitals who had less than 10% of stock of generic medicines in their hospital pharmacies, expressed a stronger agreement to the above statement when compared to the hospitals having 11%-40% or 41%-60% of generic medicines of their total stock [U=4073.50, r=−0.23, p<0.001] and [U=1126, r=−0.25, p<0.001] respectively.

Their beliefs about the low efficacy of generic medicines is further supported by another notion that the branded products are required to meet higher standards than generic medicines and 74.2% of the physicians thought in this way. This declaration was rejected by only 13.7% of the respondents. Of the respondents, 42.2% (n=111) believed that generic medicines do not have more side effects when compared to the originator products while almost 38.0% of the clinicians were in the neutral category. A significant association was found between the age of the respondents and their views on this aspect, [H(4)=18.160, p=0.001]. A Bonferroni correction at a 0.005 level of significance revealed that the physicians who were aged between 30–40 years expressed a stronger agreement to the statement when compared to those who were aged between 51–60 years [U=715.50, r=−0.24, p<0.001]. However, the opposite scenario was observed while applying a Bonferroni correction at a 0.005 level of significance, which revealed that the doctors who were aged between 41–50 years expressed a stronger agreement to the statement when compared to those who were 30–40 years old [U=1979.00, r=–0.22, p<0.001].

The majority of physicians (n=213, 81%) believed that there were too many generic medicines available and 63.1% of the respondents confirmed that the patients preferred the originator products and they got confused about generic medicines. A significant association was also observed between the ages, the years of practice, and their views on this statement. The values were [H(4)=29.647, p<0.001] and [H(5)=29.475, p<0.001], respectively. A Bonferroni correction at a 0.005 level of significance revealed that the respondents who were aged between 30–40 years expressed a stronger agreement to the statement when compared to the respondents who were between 51–60 years of age [U=711.00, r=−0.26, p<0.001]. However, the respondents who were 41–50 years of age expressed a stronger agreement to the statement when compared to the physicians who were between 30–40 years of age [U=1664.00, r= −0.31, p<0.001]. For the years of practice, a Bonferroni correction at a 0.00357 level of significance revealed that the respondents who had practised between 21–25 years expressed a stronger agreement to the statement than the physicians who had had between 6–10 years of practice [U=172.50,r=−0.20, p=0.001]. The physicians with 16–20 years of experience expressed a stronger agreement to the statement when compared to those who had been practising between 6–10 years [U=317.50, r=−0.20, p=0.001]. The prescribers with 11–15 years of practising experience expressed a stronger agreement to the statement than those who had had 21–25 years [U=652.00, r=−0.23, p<0.001] and 16–20 years of practice [U=1167.50, r=−0.25, p<0.001]. A significant association was also observed between the stock of generic medicines held in their hospital pharmacy and the statement that ‘Patients prefer original brands, they get confused with generics’ [H(3)=15.053, p=0.002]. A Bonferroni correction at a 0.0083 level of significance revealed that the doctors working within hospitals having less than 10% and 11%-40% of generic medicines in stock expressed a stronger agreement to the statement than those who had 41%-60% of generic medicines in stock [U=1237.50, r=−0.23, p<0.001] and [U=661.50, r=−0.20, p=0.001], respectively. 69.2% of the physicians confirmed that it was easier for them to remember the brand names than the generic drug product names. But, at the same time, 97.4% of the physicians appreciated that generics are cheaper than the originals. 36.9% of the physicians denied providing a liberty to the pharmacist for dispensing generic medicines and 25.9% preferred to stay neutral about this idea.

### The perceptions of the physicians towards issues pertaining to generic medicines utilisation

Tables 4 and 5 show the detailed responses of the participants regarding these issues, and the possible differences in their beliefs about generic product utilisation in Malaysia. These were assessed by testing the effects of demographic characteristics using Mann Whitney and Kruskal Wallis tests, wherever applicable. Approximately 245 (93.1%) of the respondents believed that they need standard guidelines, for both the prescribers and the pharmacists on brand substitution. 90.5% of the respondents felt that the quality use of generic medicines among patients can be achieved, if both prescribers and pharmacists work together. A similar percentage of physicians felt the need to provide more information about generic medicines to patients, in order to make sure that they understood about their medicine. A significant association was noted between the age, the years of practice and to the above statement, [H(4)=21.148, p<0.001] and [H(5)=24.263, p<0.001], respectively. A Bonferroni correction at 0.005 level of significance revealed that the doctors aged between 30–40 years expressed a stronger agreement when compared to the respondents aged between 61 to 70 years and 51 to 60 years [U=442.00, r=−0.19, p=0.002] and [U=876.00, r=−0.18, p=0.004], respectively. The response expressed by the physicians having an age from 41–50 years was stronger than the 30–40 years old physicians [U=1923.50, r= −0.26, p<0.001]. For year of graduation, Bonferroni correction at 0.005 level of significance revealed that the respondents who graduated before year 1980, from 1981–1990 and between 1991–2000 expressed stronger agreement to the statement that those who graduated between year 2006 to 2010 respectively [(U=90.00, r=−0.23, p<0.001), (U=159.5, r=−0.21, p=0.001), (U=330, r=−0.22, p<0.001)]. For years of practice, Bonferroni correction at 0.00357 level of significance revealed respondents who had longer years of practice i.e. 11–15 years, 16–20 years, 21–25 years, >25 years) expressed stronger agreement to the statement that those who had 6–10 years of practice respectively [(U=267.00, r=−0.22, p<0.001), (U=306.00, r=−0.24, p<0.001), (U=165.00, r=−0.20, p=0.001), (U=330.00, r=−0.26, p<0.001)].

**Table 4:** **Perceptions of prescribers to issues pertaining to generic medicines utilization**

**Item description**	**n (%)**				
	**Strongly disagree**	**Disagree**	**Neutral**	**Agree**	**Strongly agree**
I believe we need a standard guideline to both prescribers and pharmacist on brand substitution process	1 (0.4)	3 (1.1)	14 (5.3)	145 (55.1)	100 (38.0)
In my opinion, quality use of generic medicines among patients can be achieved if both prescribers and pharmacist work together	1 (0.4)	3 (1.1)	21 (8.0)	181 (68.8)	57 (21.7)
I think patient should be given an enough information about generic medicines in order to make sure they really understand about the medicines they take	1 (0.4)	8 (3.0)	16 (6.1)	176 (66.9)	62 (23.6)
I believe advertisement by the drug companies will influence my future prescribing pattern	1 (0.4)	66 (25.1)	87 (33.1)	97 (36.9)	12 (4.6)
I need more information on the issues pertaining to the safety and efficacy of generic medicines	0 (0.0)	8 (3.0)	12 (4.6)	158 (60.1)	85 (32.3)
Hospital budget for drug procurement factor will affect my choice of medicines	0 (0.0)	23 (8.7)	62 (23.6)	132 (50.2)	46 (17.5)

**Table 5:** **Comparison between demographic characteristic and doctors’ perceptions about issues pertaining to generic medicines utilization**

**Item description**	Gender*	Age**	Year of graduation**	Postgraduate qualification*	Country of graduation**	Year of practice**	No. of prescriptions written/day**	% of generics in stock**
I believe, we need a standard guideline to both prescribers and pharmacist on brand substitution process.	0.298	0.112	0.254	0.296	**0.002**	0.178	0.425	0.446
In my opinion, quality use of generics among patients can be achieved if both prescribers and pharmacist work together.	0.071	0.255	0.392	**0.024**	0.126	0.110	0.407	0.560
I think, patient should be given enough information about generic medicines in order to make sure they really understand about the medicines they take.	**0.022**	**< 0.001** ^**a**^	**< 0.001**	0.612	0.775	**< 0.001** ^**a**^	0.083	0.189
I believe advertisement by the drug companies will influence my future prescribing pattern.	0.072	0.315	**0.021** ^**a**^	0.289	0.726	**0.026**	0.765	**0.045**
I need more information on the issues pertaining to the safety and efficacy of generic medicines.	**0.005**	**0.007** ^**a**^	0.119	0.781	0.255	0.059	0.981	0.093
Budget for drug procurement will affect my choice of medicines.	**0.010**	**0.008** ^**a**^	**< 0.001** ^**a**^	**0.034**	0.302	**0.003** ^**a**^	1.000	0.052

*Mann Whitney test.

**Kruskal Wallis test was used.

P < 0.05 is considered significant.

^**a**^Significant after Bonferroni correction. Bold values indicate statistical significance.

The physicians who needed more information about the safety and the efficacy of generic medicines constituted almost 92.4% of the total sample studied. An age-related significant association [H(4)=14.077, p=0.007] was observed about this aspect. Bonferroni correction at 0.005 level of significance revealed that the respondents aged between 41–50 years old expressed a stronger agreement to the statement when compared to the physicians who were aged between 30–40 years (U=2017.50, r=−0.24, p<0.001). Only 41.5% of the respondents agreed that drug advertisements by the manufacturers would influence their prescribing behaviour and a significance association was observed among year of graduation [H(4)=11.508, p=0.021]. Bonferroni correction at 0.005 level of significance revealed that the physicians who graduated between the years 1991–2000 expressed a stronger agreement to the statement that advertisements by the drug companies would influence their future prescribing patterns. This agreement was stronger when compared to the physicians who had graduated from 2006–2010 (U=339.00, r=−0.19, p=0,002).

When the respondent’s views were sought concerning their budgets for drug procurement versus their prescription decision, 67.7% of the physicians agreed that it would affect their choice of medicine. Significant associations [H(4)=13.897, p=0.008], [H(4)=20.526, p<0.001] and [H(5)=17.834, p=0.003] were observed between their age, year of graduation, experience, and the physician’s responses respectively, when replying to the statement about pharmaceuticals procurement budgets. Bonferroni correction at 0.005 level of significance revealed that the physicians in the age group of 41–50 years expressed a stronger agreement to the statement when compared to age group of 30–40 years (U=2179.00, r=−0.18, p=0.004). Similarly, the respondents who graduated between the years 1991–2000 expressed a stronger agreement to the statement when compared to the doctors who graduated between the years 2001–2005 (U=445.50, r=−0.184, p=0.003, at 0.005 level of significance for Bonferroni correction). Further, the Bonferroni correction at 0.00357 level of significance showed that the doctors who were practising between 16–20 years (U=303.50, r=−0.21, p=0.001) and 21–25 years (U=171.00, r=−0.19, p=0.002) expressed a stronger agreement to the statement when compared to those physicians who had had 6–10 years of experience.

## Discussion

The response rate achieved in this survey was 35.8%. Generally, it is more exigent to get a high response rate from the surveys of the physicians than from the general population surveys [33,34]. Parson et al. reported that follow up attempts, up to 11 times in the mail surveys of physicians, yielded less than 20% of a response when compared to the first mailing, which accounted for up to 40% of a response [33]. Moreover, it is known that it is more challenging to obtain high response rates especially from physicians practicing in private healthcare sector in Malaysia [29,35,36]. Hence, considering the cost and time, only two attempts were made to each of the hospitals. More importantly, it has been reported that, although changing, physicians remained a relatively homogenous population with regard to their knowledge, training, attitudes and behavior [37,38].

The majority of the physicians who participated in this survey indicated a very low generic medicine prescription rate. They preferred to use originator drug products and write their prescriptions using brand names. This finding is consistent with the findings reported in a qualitative study by Kumar et al. [20]. This phenomenon was observed in all of the age groups and was independent of their year, their place of graduation and experience. However, in a study conducted by Chua et al. which explored the knowledge and perceptions of general practitioners (GPs) concerning the use of generic medicines in Penang, Malaysia reported that prescribing generic medicines was much more apparent among GPs with a practice of less than 30 years. It was also reported that the country of graduation influenced a respondent’s prescribing trend [29]. In our study, it was interesting to note that physicians with postgraduate degree tended to prescribe more originator products than generic medicines than those physician with only the basic medical degree (28.3% versus 18.4% respectively wrote their prescriptions by always using a brand name product). In Chua et al.’ study, approximately 85% of GPs were actively prescribing generic medicines. The higher use of generic medicines among the GPs can be correlated to the high mark ups in the cost of the medicines, as dispensed by these doctors, in order to make more profit [16]. Generic medicines, being cheaper, provide more opportunity to be sold at a higher price, since there is no drug price control in Malaysia [17,18]. However, in private medical centres, physicians tend to prescribe branded products since the profit from the sale of medicines in these hospitals does not come to the physicians. This is different from the situation in GP clinics because in GPs clinics the profit goes directly to them as they are the owners of these clinics. Thus, profitability of the product is more influencing factor for GPs rather than physicians from private medical centres. In this study, we can infer that irrespective of age category, experience, and the place of graduation, the physicians working in the private medical centres of Malaysia prefer to use branded medicines in their practice, and their conduct is affected, depending on their knowledge, their beliefs and their perceptions about generic medicines.

In the qualitative study by Kumar et al., it was reported that the physicians interviewed had a general idea about bioequivalence [20], but this quantitative study provides further insight in this topic. 97.7% of the physicians were unaware of the bioequivalence criteria for generic medicines set by the Malaysian Regulatory Agency i.e. the NPCB. This finding is similar to those reported from other countries where the physicians had a poor knowledge of the bioequivalence acceptability criteria set by their respective drug regulatory agencies [26,39]. Hence, both the qualitative and quantitative studies, involving physicians from the private medical centres in Malaysia, revealed that the respondents have a rough idea about bioequivalence, and its role in the generic industry, but they lacked a detailed knowledge about this subject. Surprisingly, 51.3% of the respondents considered that generic products are bioequivalent, but still 74% (combining both always and usually categories) prefer to write their prescriptions using brand names. This can be correlated to the statement given by the physicians in the above quoted qualitative study, about the fact that the “GPs have been using generic medication and the patient did not get the required relief. Now, he or she (the patient) has come to a private hospital, and we change the medication, use originators, because we prefer to give them better treatment” [20]. In addition, 74.2% of the physicians assumed that the manufacturing and the quality control standards of branded products are much higher than generic medicines. 51.7% of the respondents stated that generic medicines are less effective when compared to their branded counterpart. The results of such perceptions are also depicted from the availability of very low stock (<10%) of generic medicines in the pharmacies of their hospitals. A typical trend was observed in the stock of generic medicines and their belief in the statement that generic medicines have a low efficacy when compared to branded products (less than 10% of generic stock > 11-40% > 41-60%). 81% of the physicians thought that there are too many generic brands available and 63.1% of them have commented that it leads to confusion among patients. The lack of understanding about the regulatory requirements of generic drug manufacturing, and these perceptions, can have a negative impact on generic medicine prescribing [29]. Thus, the findings of this study indicate a strong need for awareness or educational programmes regarding generic medicines. It is of utmost importance that generic medicine awareness should not only be imparted to consumers, but also to the prescribers. The awareness campaigns coordinated by NPCB with focus on bioequivalence and cGMP requirements for generic medicines will be of great help.

The participating physicians also expressed the need for a standard guideline on brand substitution. The implementation of such a guideline, for both physicians and pharmacists, on generic substitution, would further encourage the use of generics, and maintain the accessibility and the affordability of medicines [40]. Generic substitution policies, in many countries, allow the pharmacists to dispense a different brand of the drug, even when the prescription is written for a particular brand [41]. The implementation of the Malaysian National Medicine Policy [42] and the nationwide policy of substituting originator or patented drugs with generic medicines, at government hospitals, are a step forward taken by the Malaysian government; but its healthcare system still lacks actual generic substitution policies, which should be implemented in all kinds of medical facilities including the private sector [18]. The surveyed physicians also agreed that it was important to establish a greater collaboration between them and the pharmacists, in order to improve generic utilisation among consumers. They also articulated the need to provide more information to the patients about generic medicines. This complements the efforts of the Malaysian government to spread generic medicine awareness through nationwide road shows, as a cost containment strategy [20,32]. Previous studies conducted in Malaysia have also revealed that the majority of consumers do not have a fair idea about generics [43]. Hence, high end educational interventions can potentially augment the usage of generic medicines in the private medical hospitals, by reaching both the physicians and the consumers. Studies have revealed that educational interventions on generic drugs have increased their utilisation by medicine consumers [44]. Efforts should be made to educate and to persuade physicians, in the early stage of their career, about the benefits and the value of generic medicine prescribing [25,32,45]. An increased knowledge of consumers about generic medicines can also help them to request generic medicine when they visit such medical centres. This might result in an improved adherence and significant savings to the OOPPs [20,46]. A significant number of participants agreed that their selection of medicine was influenced by drug advertising, but the advertising should be based on hard evidence or a study. The physicians wished to see a bioequivalence report for generic medicines, as evidence, when the medical representatives from the generic companies come to promote their product [20]. This is a genuine request, since cost is a secondary factor for the private hospital physicians, and generally, a cheaper product is the only focus for promotion by the generic manufacturers. Other published literatures have also reported that the prescription behaviour of physicians is affected by their interactions with the pharmaceutical manufacturers [47,48]. The physicians involved in this survey also requested for more information on the issues pertaining to the safety and the efficacy of generic medicines. This information is necessary to boost their confidence in generic medicines. A medical representative is the first source of information for the physician [25] who should not only be able to discuss cost, but should also explain about the available bioequivalence, stability, use of ‘generally regarded as safe’ (GRAS) listed excipients and cGMP processes involved in the manufacturing of his company’s product. The budgets to procure medicines, or the patient’s socio-economic condition, are supposed to be the major factors for generic substitution in the private hospitals in Malaysia, which was also identified in a previous qualitative study [20].

The findings from this survey suggest it is important that the authorities and heads of the medical profession in Malaysia take steps to enhance prescribing efficiency building on examples in the literature [49-53]. For instance, in Europe, several measures and interventions have been undertaken to enhance prescribing of generic medications. These measures particularly the initiatives and interventions that aim to enhance prescribing and dispensing of generic medications are collated under “4 Es”, i.e. education, engineering, economics and enforcement [49-51]. The implementation of these measures has successfully promoted generic medications in Europe. Therefore, Malaysia can learn from these experiences. For example, beside distribution of educational printed material, more intensive strategies such as academic detailing and monitoring of prescribing behavior coupled with feedback can be adopted in Malaysia. Moreover, development of a list of non-interchangeable medications to address the concern of bioequivalence and therapeutic equivalence is recommended in the literature [53,54]. Furthermore, international non-proprietary name (INN) prescribing is one of the measures adopted in several countries to promote generic medications (e.g. in the UK, Lithuania and Estonia) [50]. This is important given that INN prescribing is currently not a common practice in Malaysia [55]. More importantly, from other countries experiences, it is evident that a combination of measures is needed to promote utilization of generic medicines rather than a single measure [49,51,54]. Moreover, the promotion efforts need to target all the involved parties including physicians, pharmacists and patients [56].

### Limitations of the study

The present study is subjected to the limitations that are relevant to surveys where postal questionnaires are used as a tool to extract data [57]. In particular, the low response rate might influence generalization of the findings to the whole population. However, it is very similar to all previous studies conducted with physicians in private sector in Malaysia [29,35,36]. Additionally, the representativeness of the respondents’ features and responses could not be fully evaluated, since no national data on the demographic and practice characteristics of the physicians working within the private medical centres in Malaysia is available. Overall, we believe that this study is valuable, in providing a suggestive national baseline data on the Malaysian private healthcare sector, physician’s perceptions, and the practices regarding generic medicines.

## Conclusion

The majority of the physicians from private medical hospitals in Malaysia who participated in this survey indicated a low generic medicine prescription rate. They preferred to use originator drug products and write their prescriptions using brand names. This phenomenon was observed in all of the age groups and was independent of their year, their place of graduation and experience. The respondents had negative perceptions about safety, quality and the efficacy of generic medicines which could have resulted in limited use of generic medicines in private hospitals in Malaysia. In order to facilitate wide use of generic medicines, it is recommended that the physicians need to be reassured, and educated about the drug regulatory authority’s approval system, especially with regard to bioequivalence, quality and safety. Such interventions need to be planned, in support of, and in corroboration with the NPCB, to make them more effective.

Apart from the policy on generic substitution, it would also be desirable to have a national medicine pricing policy, which controls drug prices, in both the public and private sector. These efforts are worthwhile to reduce the drug expenditure and improve the medicine affordability in Malaysia.
